# Salvage Pelvic Lymph Node Dissection After Fluciclovine Positron Emission Tomography/Computed Tomography Detected Prostate Cancer Recurrence

**DOI:** 10.1089/cren.2018.0011

**Published:** 2018-04-01

**Authors:** Madeline Cancian, Jorge Pereira, Joseph F. Renzulli

**Affiliations:** ^1^ Department of Urology, Warren Alpert Medical School, Brown University, Providence, Rhode Island.; ^2^ Minimally Invasive Urology Institute, Department of Urology, The Miriam Hospital, Providence, Rhode Island.

**Keywords:** prostate cancer, metastatic prostate cancer, metastasis-directed therapy, robotic pelvic lymph node dissection

## Abstract

***Background:*** Multiple new systemic agents have been targeted to metastatic prostate cancer, with decreased progression of disease but no cure. Surgical management of metastatic disease has been gaining interest, primarily in the setting of high-risk prostatectomies. However, metastasis-directed surgical intervention has been employed in rare scenarios, especially in oligometastatic disease. We report here on a salvage robot-assisted pelvic lymph node dissection for a solitary metastatic site.

***Case Presentation:*** A 63-year-old Hispanic man who was initially treated with prostatectomy for intermediate risk cancer developed rapid biochemical recurrence. After salvage radiation, fluciclovine positron emission tomography (PET)/computed tomography (CT) scan showed a solitary pelvic lymph node metastasis. A robot-assisted laparoscopic pelvic lymph node dissection was carried out, with subsequent nadir of his prostate-specific antigen at 0.026.

***Conclusion:*** To our knowledge, this is the first report of salvage pelvic lymph node dissection after metastatic detection by fluciclovine PET/CT scan. Our patient experienced a complete biochemical response; however, it remains to be seen whether this will be a lasting response. Surgical resection of metastatic sites in prostate cancer offers a safe alternative to systemic therapy and avoids systemic side effects.

## Introduction

Metastatic prostate cancer has traditionally been managed medically. Multiple new agents have been developed that target metastatic sites and slow down the progression of prostate cancer, but offer no hope at cure. Recently there has been interest in surgical treatment of advanced prostate cancer. Prostatectomies for high-risk disease, and salvage prostatectomies after failed radiation therapy, are starting to gain support within the urologic community. More accurate imaging, especially through new modalities within positron emission tomography/computed tomography (PET/CT), has allowed earlier identification of metastatic sites of cancer. This new technology, paired with an evolving treatment paradigm, opens the door for aggressive surgical management. Here, we discuss a case of advanced prostate cancer managed with a metastasectomy.

## Presentation of Case

Our patient was a healthy 63-year-old man who presented in December, 2014, with an elevated prostate-specific antigen (PSA) of 5.6. He underwent a prostate biopsy and was found to have T1c prostate cancer, Gleason 3 + 4 in 4 of 12 cores. In February 2015, he underwent a robot-assisted laparoscopic radical prostatectomy, which showed T3a Gleason 4 + 3 with tertiary pattern 5 disease with negative margins. His PSA nadir was 0.03; however, it was found to rise to 0.1 at 9 months. He underwent staging imaging, including a pelvic MRI and bone scan, which were negative for metastatic disease or radiographic local recurrence. After meeting with a radiation oncologist, he underwent salvage intensity-modulated radiation therapy. At this time, he was offered a short course of androgen deprivation therapy; however, he refused any systemic therapy, feeling that the side effect profile outweighed the potential therapeutic benefit. His postsalvage radiation PSA nadir was 0.09; however, it quickly rose to 0.389 with a PSA doubling time of 5 months.

**FIG. 1.**
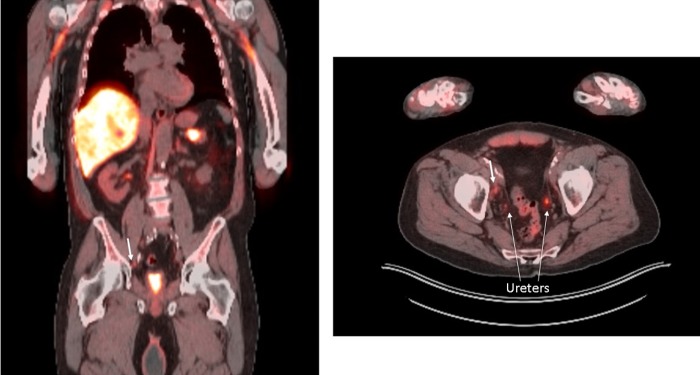
Flucicolvine PET/CT scan showing 1.1 × 0.5 cm mildly enlarged *right* pelvic sidewall lymph node with focally increased uptake suspicious for metastatic prostate cancer. The *thick white arrows* point to site of positive lymph node. *Thin white arrows* point to ureters. PET = positron emission tomography.

At this time, he elected to undergo a fluciclovine PET/CT, with findings of a right nonenlarged pelvic sidewall lymph node with increased intensity of uptake compared with surrounding tissue (Fig. 1). Bone scan at that time was negative. He underwent a robot-assisted laparoscopic bilateral pelvic lymph node dissection. Intraoperatively, there were bilateral grossly enlarged lymph nodes, and final pathology report returned 2 of 14 lymph nodes positive for metastatic prostate cancer. He recovered well, and repeat PSA at 6 weeks was 0.026.

## Discussion and Literature Review

Fluciclovine, a synthetic amino acid analogue for leucine, is increasingly being used as a tracer to identify metastatic sites of prostate cancer. It gained Food and Drug Administration approval for imaging in suspected prostate cancer recurrence in May of 2016. Its main strengths lie in its widespread availability and its ability to detect metastases at low PSA levels. Unlike choline PET/CT, fluciclovine PET/CT does not require any special equipment and thus can be performed in any nuclear medicine department. In patients with biochemical recurrence after primary treatment for prostate cancer, flucicolovine scans detected at least one site of metastatic disease in 41.4% of patients with a PSA <0.79 ng/cc and >70% of men with a PSA <6 ng/cc.^1^

With the ability to detect metastases much earlier in the disease process, it leaves practitioners in a query as to what to do with this information. These patients would traditionally be classified as having biochemical recurrence, with treatment options based primarily on type of primary treatment, or nonmetastatic prostate cancer, with limited treatment options. With very early diagnosed sites of metastatic disease, patients are now eligible for targeted therapies such as docetaxel, abiraterone, or enzalutamide. However, there is no evidence that starting these agents earlier in the disease process prolongs progression-free survival.

An alternative approach is to initiate metastasis-directed therapy (MDT). Radiation therapy has been targeted with advanced imaging to metastatic sites, either nodal, bone, or visceral; however, these studies have been small and without an adequate control group to compare oncologic outcomes.^2^ Metastatic-directed pelvic and retroperitoneal lymph node dissections after a positive choline PET/CT have been reported with initial complete biochemical response (PSA <0.2 mg/cc) in 46% of patients.^3^ In this cohort of patients, who were also treated with salvage radiotherapy, 25.6% had clinical progression-free survival at 5 years. A recent phase 2 prospective randomized trial comparing surveillance versus MDT for oligometastatic disease showed improved androgen-deprivation therapy free survival in the MDT group.^4^

Although these advances in therapy are encouraging, it remains to be seen whether there is any progression-free survival, or overall survival benefit, in patients with early detected metastases who receive early MDT.

## Conclusion

To our knowledge, this is the first report of salvage pelvic lymph node dissection after metastatic detection by fluciclovine PET/CT scan. Our patient experienced a complete biochemical response; however, it remains to be seen whether this will be a lasting response. Surgical resection of metastatic sites in prostate cancer offers a safe alternative to systemic therapy and avoids systemic side effects.

## Disclosure Statement

M.C. and J.P. have no competing financial interests. J.R. is a primary investigator at ENACT trial, SPARTAN trial, and ARAMIS trial. He is also speaker/consultant for Astellas/Medivation, Bayer, and Janssen.

## Abbreviations Used

CT: computed tomography
MDT: metastasis-directed therapy
MRI: magnetic resonance imaging
PET: positron emission tomography
PSA: prostate-specific antigen
